# The value of [^11^C]-acetate PET and [^18^F]-FDG PET in hepatocellular carcinoma before and after treatment with transarterial chemoembolization and bevacizumab

**DOI:** 10.1007/s00259-017-3724-2

**Published:** 2017-05-29

**Authors:** Shuren Li, Markus Peck-Radosavljevic, Philipp Ubl, Wolfgang Wadsak, Markus Mitterhauser, Eva Rainer, Matthias Pinter, Hao Wang, Christian Nanoff, Klaus Kaczirek, Alexander Haug, Marcus Hacker

**Affiliations:** 1 Department of Biomedical Imaging and Image-guided Therapy, Division of Nuclear Medicine, Medical University of Vienna, Vienna, Austria; 2 Department of Internal Medicine III, Division of Gastroenterology and Hepatology, Medical University of Vienna, Vienna, Austria; 3 Department of Nuclear Medicine, Peking Union Medical College Hospital, Chinese Academy of Medical Sciences, Peking Union Medical College, Beijing, China; 4 Institute of Pharmacology, Medical University of Vienna, Vienna, Austria; 5 Department of Surgery, Medical University of Vienna, Vienna, Austria

**Keywords:** Hepatocellular carcinoma, FDG, Acetate, Pet, Bevacizumab, Transarterial chemoembolization

## Abstract

**Purpose:**

This prospective study was to investigate the value of [^11^C]-acetate PET and [^18^F]-FDG PET in the evaluation of hepatocellular carcinoma (HCC) before and after treatment with transarterial chemoembolization (TACE) and vascular endothelial growth factor (VEGF) antibody (bevacizumab).

**Methods:**

Twenty-two patients (three women, 19 men; 62 ± 8 years) with HCC verified by histopathology were treated with TACE and bevacizumab (*n* = 11) or placebo (*n* = 11). [^11^C]-acetate PET and [^18^F]-FDG PET were performed before and after TACE with bevacizumab or placebo. Comparisons between groups were performed with t-tests and Chi-squared tests, where appropriate. Overall survival (OS) was defined as the time from start of bevacizumab or placebo until the date of death/last follow-up, respectively.

**Results:**

The patient-related sensitivity of [^11^C]-acetate PET, [^18^F]-FDG PET, and combined [^11^C]-acetate and [^18^F]-FDG PET was 68%, 45%, and 73%, respectively. There was a significantly higher rate of conversion from [^11^C]-acetate positive lesions to negative lesions in patients treated with TACE and bevacizumab as compared with that in patients with TACE and placebo (*p* < 0.05). In patients with negative acetate PET, the mean OS in patients treated with TACE and bevacizumab was 259 ± 118 days and was markedly shorter as compared with that (668 ± 217 days) in patients treated with TACE and placebo (*p* < 0.05). In patients treated with TACE and placebo, there was significant difference in mean OS in patients with positive FDG PET as compared with that in patients with negative FDG PET (*p* < 0.05). The HCC lesions had different tracer avidities showing the heterogeneity of HCC.

**Conclusions:**

Our study suggests that combining [^18^F]-FDG with [^11^C]-acetate PET could be useful for the management of HCC patients and might also provide relevant prognostic and molecular heterogeneity information.

## Introduction

Hepatocellular carcinoma (HCC) is one of the most malignant neoplasms [1, 2]. The incidence of HCC is still growing [1, 2]. Early detection of HCC may play the most important role for the prognosis of HCC [1, 2]. Usually, thorough staging is performed, including computed tomography (CT), abdominal ultrasound (US), and magnetic resonance imaging (MRI) [2]. However, each of the above-mentioned methods has limitations. [1, 3]. For many patients who have non-resectable, intermediate stage HCC, transarterial chemoembolization is recommended as the preferred treatment option [4, 5]. Several studies have demonstrated the survival benefit of chemoembolization [5]. It has been demonstrated that vascular endothelial growth factor (VEGF) overexpression is a prognostic indicator of poor survival in patients with HCC [6]. VEGF is further up-regulated immediately after chemoembolization, and VEGF levels after treatment are an independent predictor of tumor response and survival [7]. Bevacizumab is a humanized murine monoclonal antibody targeting VEGF and blocks its binding to its receptors, thereby preventing the formation of new blood vessels and inhibiting the growth of existing tumors and metastases [8]. It has been demonstrated that the treatment with bevacizumab in patients with HCC may lead to prolonging of life time with approximately six-month stability in patients with previous rapid tumor growth [9].

Positron emission tomography (PET) using [^18^F]-FDG has been applied successfully in detecting many malignant diseases; however, it has a high false negative rate of approximately 40–50% in the detection of HCC (10). Some studies [10–12] have shown that [^11^C]-acetate PET is useful in the localization of HCC lesions. Up to now, no data are available on the comparison between [^11^C]-acetate PET and [^18^F]-FDG PET in the evaluation of therapy response in patients with HCC after treatment with bevacizumab, and the relation of [^18^F]-FDG PET or/and [^11^C]-acetate PET findings towards survival is not quite known. Based on our previous study [11], we prospectively investigated HCC patients in this study with the following purposes: (1) to evaluate the role of [^11^C]-acetate PET and [^18^F]-FDG PET in the staging of HCC; and (2) to evaluate the therapy response by using [^11^C]-acetate PET and [^18^F]-FDG PET, as well as (3) to assess whether [^18^F]-FDG PET or/and [^11^C]-acetate PET predict survival in HCC patients after the treatment with TACE and bevacizumab or placebo.

## Materials and methods

### Patients

This study was part of the clinical study that was approved by the ethical board of the Medical University of Vienna and is registered at ClinicalTrials.gov (ClinicalTrials.gov Identifier: NCT00280007) [13].

All patients were treated using conventional TACE. TACE was carried out with doxorubicin (Pfizer, 75/50/25 mg/m^2^) mixed with lipiodol (1:1) in a total volume of 20 mL, followed by particle embolization with an embolic agent (Bead Block; Biocompatibles, UK) [14]. Patients were randomized to the bevacizumab group, which received bevacizumab in masked bottles (Avastin®, Roche Austria, Vienna; 5 mg/kg) intravenously prior to the first TACE (same day) and every 14 days thereafter for 52 weeks or until one of the following events occurred: patient death, occurrence of extrahepatic lesions, or untreatable tumor progression. Patients randomized to the control group received a saline infusion in identically masked bottles at the same time points as the bevacizumab-treated patients. After the first TACE procedure was completed, TACE was repeated twice at 4-week intervals.

**Fig. 1:**
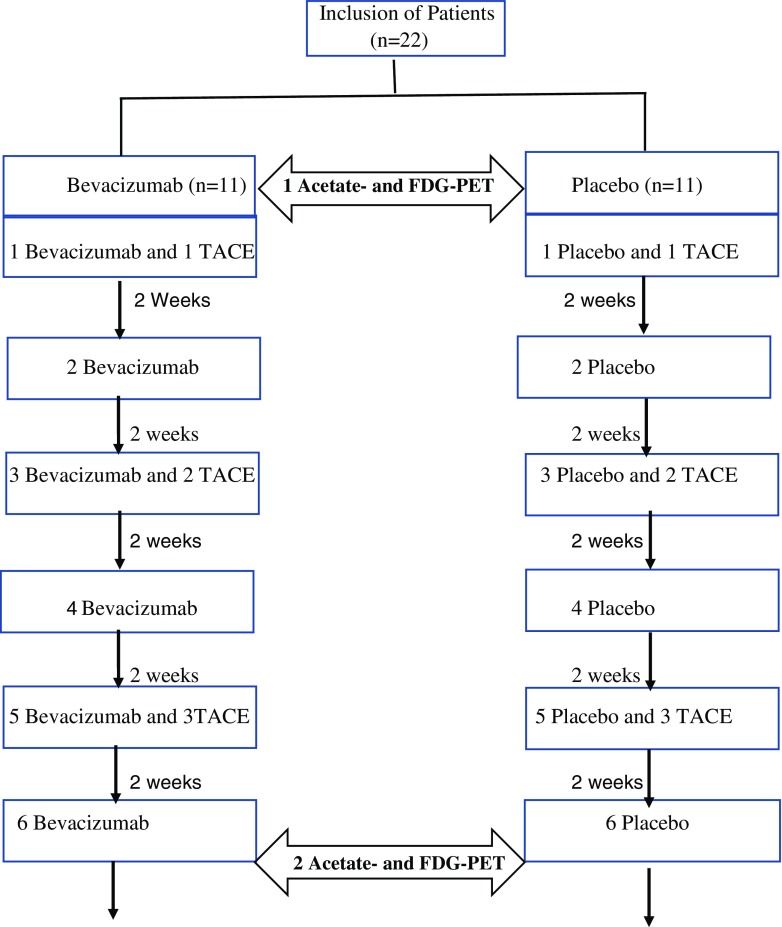
Flow diagram

All patients underwent [^11^C]-acetate PET and [^18^F]-FDG PET within one day (seven patients) or two days (14 patients) for comparison prior to the treatment (first PET-examinations) and after treatment with three cycles of TACE and six cycles of bevacizumab or placebo (second PET-examinations). Figure 1 showed the flow diagram of this study.

### [^11^C]-acetate PET

[^11^C]-acetate was synthesized according to our previous publication [15].

All patients underwent PET imaging in the supine position after a 6 h fast. A dedicated PET system (GE Advance, General Electric Medical Systems, Milwaukee, WI, USA) was used. Emission scans from mid-thigh to skull base were acquired 10 min after intravenous injection of 8 MBq of [^11^C]-acetate per kg of body weight with 5 min per scanner bed position. PET images were reconstructed using ordered subset expectation maximization (OSEM) with all relevant corrections applied.

### [^18^F]-FDG pet

The same whole body PET scanner (GE Advance, General Electric Medical Systems, Milwaukee, WI, USA) was used. The patients had been fasted for at least 4 h before the injection of [^18^F]-FDG PET. [^18^F]-FDG PET was administered intravenously in a dosage of 5.5 MBq/kg of body weight in patients. Subsequently, the acquisition of whole body images started 50 min later. Emission and transmission scans were performed in a two-dimensional imaging method for data acquisition. While emission images were acquired for 3 min per bed position, each post-emission transmission scan was obtained for 1 min per bed position; whole-body scanning was performed from skull base to upper thigh in all patients using five or six bed positions according to the height of each patient. Reconstruction of the data was performed by using the ordered subset expectation method (OSEM) with 16 subsets, three iterations and 128 × 128 matrix (pixel) size.

### Image analysis

[^11^C]-acetate PET and [^18^F]-FDG PET were applied within 1 day (*n* = 7) or 2 days (*n* = 15) before the treatment with TACE and bevacizumab. Follow-up PET examinations were done after the treatment with three cycles of TACE and six cycles of bevacizumab or placebo. The PET pictures were interpreted both visually and semiquantitatively for the regions with pathologic tracer accumulation using standardized uptake value (SUV). With the assistance of a 3-point grading system (isometabolic, hypermetabolic, and hypometabolic) the intrahepatic primary lesions were interpreted visually. The system compared data with tracer uptake by normal liver parenchyma for [^11^C]-acetate PET and [^18^F]-FDG PET. A lesion was assumed to be a malignant hepatic mass, if it was hypermetabolic on at least one image from [^11^C]-acetate PET or [^18^F]-FDG PET.

Scintigraphic results with [^11^C]-acetate PET and [^18^F]-FDG PET were directly compared and evaluated in each patient against those of recent CT and/or MRI, ultrasonographic imaging, bone scintigraphy, and surgical exploration with consecutive histological analysis. When scan results of [^11^C]-acetate PET and [^18^F]-FDG PET corresponded with those of the above-mentioned conventional imaging methods or when a corresponding lesion appeared on conventional imaging during the follow-up period, scan lesions were rated as true-positive. Lesions not detected on scan, but seen on conventional imaging and showing progression during the follow-up period or confirmed by histological examination were rated as false-negative. Scan results suggestive for tumor lesions without corresponding lesions found on conventional imaging within the follow-up period were rated as false-positive. The follow-up of the 22 patients investigated ranged from 6 to 14 (mean 7.8) months. The ROI was drawn manually around the tumor. The same size and shape ROI was placed on the non-tumor liver tissue in the same patient as a reference. For the patients with multifocal or diffuse disease, the ROI was drawn either automatically or manually around the representative lesions or areas. In some of these patients with an infiltrative disease, correlative CT or MRI scans and PET imaging were available to aid in locating non-tumor liver tissue.

Response to treatment included functional imaging with [^11^C]-acetate for assessment of the acetate metabolic response (AMR) and [^18^F]-FDG for FDG metabolic response (FMR), as well as the assessment according to RECIST criteria [16].

### Statistical analysis

Comparisons between groups were performed with t-tests and Chi-squared tests, where appropriate. Overall survival (OS) was defined as the time from start of bevacizumab or placebo until the date of death/last follow-up, respectively. OS was calculated by the Kaplan-Meier method. Univariate analyses were performed by means of Cox regression. Correlation studies were evaluated by linear regression analysis and the Pearson or Spearman correlation analysis, where appropriate. A *p* value <0.05 was considered statistically significant. All statistical analyses were performed using Sigma Plot version 11.0 (Systat Software Inc., CA, USA).

## Results

### Patients

From January 2006 to December 2009, a total of 32 patients with histologically confirmed HCC in early or intermediate stage (BCLC A or B) were included with written informed consent. Among them, complete [^18^F]-FDG PET and [^11^C]-acetate PET data were not available for 10 patients and were not included in the statistical analysis. Therefore, a total of 22 patients (19 men, three women; 62 ± 8 years) were investigated for statistical analysis.

Twenty-two patients enrolled in the study had HCC, 70 HCC tumor nodules were detected in total. For 22 patients, HCC was examined by histopathology by percutaneous biopsy. The histological grade was well differentiated for seven patients (32%), moderately differentiated for 11 (50%), and poorly differentiated for four (18%). The baseline and histopathological characteristics of the patients are summarized in Table 1.

**Table 1:** Baseline and histopathological characteristics of 22 patients with HCC

Features	No. of Patients (*n* = 22)	TACE + Avastin (*n* = 11)	TACE + Placebo (*n* = 11)
Age in years (range)	62 ± 8 (47–78)	63 ± 7 (51–78)	61 ± 7 (47–75)
Sex (n)			
Male	19	9	10
Female	3	2	1
Etiology of liver disease			
Hepatitis C	11	4	7
Alcoholism	8	4	4
Other	3	3	0
Child–Pugh Class			
A	18	9	9
B	4	2	2
WHO performance			
0	17	7	10
1	4	3	1
2	1	1	0
Tumor number (n)			
1	5	3	2
2	4	3	1
3	3	2	1
4	3	1	2
> 4	7	2	5
BCLC (n)			
A	4	2	2
B	18	9	9
Max tumor size			
Median cm (range)			
< 3 (0.5–2.5)	4	2	2
≥ 3 (3.0–9.5)	18	9	9
Differentiation status			
well differentiated (G1)	7	3	4
moderately differentiated (G2)	11	7	4
poorly differentiated (G3)	4	1	3

### [^11^C]-acetate PET and [^18^F]-FDG PET for detecting HCC before treatment

The sensitivity of [^18^F]-FDG and [^11^C]-acetate for detecting the 70 HCC lesions in the 22 patients is shown in Table 2.

**Table 2:** Comparison of scan results, tumor size, and therapy (metabolic) response between patients treated with TACE plus bevacizumab and patients with TACE plus placebo

	TACE + Bevacizumab	TACE + Placebo	Total
Sensitivities before the treatment (%)			
patient-related Acetate PET	7/11 (64)	8/11 (73)	15/22 (68)
patient-related FDG PET	4/11 (36)	6/11 (55)	10/22 (45)
lesion-related Acetate PET	16/32 (50)	20/38 (53)	36/70 (51)
lesion-related FDG PET	4/32 (13)	12/38 (32)	16/70 (23)
patient-related Acetate PET + FDG-PET	7/11 (64)	9/11 (82)	16/22 (73)
lesion-related Acetate PET + FDG-PET	16/32 (50)	24/38 (63)	40/70 (57)
Acetate PET in well differentiated HCC	2/3 (67)	3/4 (75)	5/7 (71)
Acetate PET in moderately differentiated HCC	4/7 (57)	2/4 (50	6/11 (55)
Acetate PET in poorly differentiated HCC	1/1 (100)	3/3 (100)	4/4 (100)
FDG PET in well differentiated HCC	1/3 (33)	2/4 (50)	3/7 (43)
FDG PET in moderately differentiated HCC	2/7 (29)	1/4 (25)	3/11 (27)
FDG PET in poorly differentiated HCC	1/1 (100)	3/3 (100)	4/4 (100)
Tumor size^a^ before the treatment (cm)	5.9 ± 5.1	6.3 ± 5.6	6.1 ± 5.5
Therapy (metabolic) response after the treatment (%):			
patient related AMR	6/7 (86)	3/8 (38)	9/15 (60)
lesion related AMR	14/16 (88) *	6/20 (30)	20/36 (56)
patient related FMR	2/4 (50)	2/6 (33)	4/10 (40)
lesion related FMR	2/4 (50)	3/12 (25)	5/16 (31)
Tumor size^a^ after the treatment (cm)	2.8 ± 1.5**	3.1 ± 2.9**	3.0 ± 1.9**

Data in parentheses are percentages; * *p* < 0.05 versus group TACE +Placebo; ** *p* < 0.05 versus tumor size before the treatment; ^a^ Longest diameter was measured.

AMR: the rate of conversion from ^11^C–acetate-PET-positive to negative. FMR: the rate of conversion from ^18^F–FDG-PET-positive to negative.

Fifteen patients (68%) had [^11^C]-acetate-positive lesions; ten patients (45%) had [^18^F]-FDG-positive lesions. Thus, 16 of the 22 patients with HCC (73%) had lesions that were scored positive for [^11^C]-acetate and/or for [^18^F]-FDG, and six patients had both [^11^C]-acetate-negative and [^18^F]-FDG-negative lesions. Among seven [^11^C]-acetate-negative patients, six patients were [^18^F]-FDG-negative, and among 12 [^18^F]-FDG-negative patients there were six patients with negative [^11^C]-acetate scan results.

In a lesion-per-lesion analysis, the sensitivity of PET for the detection of HCC was 51% (36/70) with [^11^C]-acetate, 23% (16/70) with [^18^F]-FDG and 57% (40/70) when both tracers were combined.

71% (5/7) of patients with well differentiated tumors were positive for [^11^C]-acetate and 43% for [^18^F]-FDG (3/7), whereas only 27 % (3/11) of patients with moderately differentiated HCC were positive for [^18^F]-FDG and 55% for [^11^C]-acetate (6/11). Interestingly, all four patients with poorly differentiated tumor in this study was positive for both [^18^F]-FDG (100%) and [^11^C]-acetate (100%). In patients with [^11^C]-acetate positive and [^18^F]-FDG-negative tumors were moderately or well differentiated tumors.

### [^11^C]-acetate PET and [^18^F]-FDG PET for evaluation of therapy response

The therapy (metabolic) response of the 70 HCC lesions in the 22 patients after the treatment by using [^18^F]-FDG and [^11^C]-acetate is demonstrated in Table 2.

The rate of conversion from [^11^C]-acetate positive to negative (i.e. AMR) was 38% in patients treated with TACE and placebo and 86% in patients with TACE and bevacizumab. The rate of conversion from [^18^F]-FDG PET positive to negative (i.e. FMR) was 33% in patients treated with TACE and placebo and 50% in patients with TACE and bevacizumab.

In a lesion-per-lesion analysis, the rate of conversion from [^11^C]-acetate positive lesions to negative lesions (i.e. AMR) was 30% in patients treated with TACE and placebo and 88% in patients with TACE and bevacizumab. There was a statistically significant difference (*p* = 0.043). The rate of conversion from [^18^F]-FDG PET positive lesions to negative lesions (i.e. FMR) was 25% in patients treated with TACE and placebo and 50% in patients with TACE and bevacizumab.

**Fig. 2:**
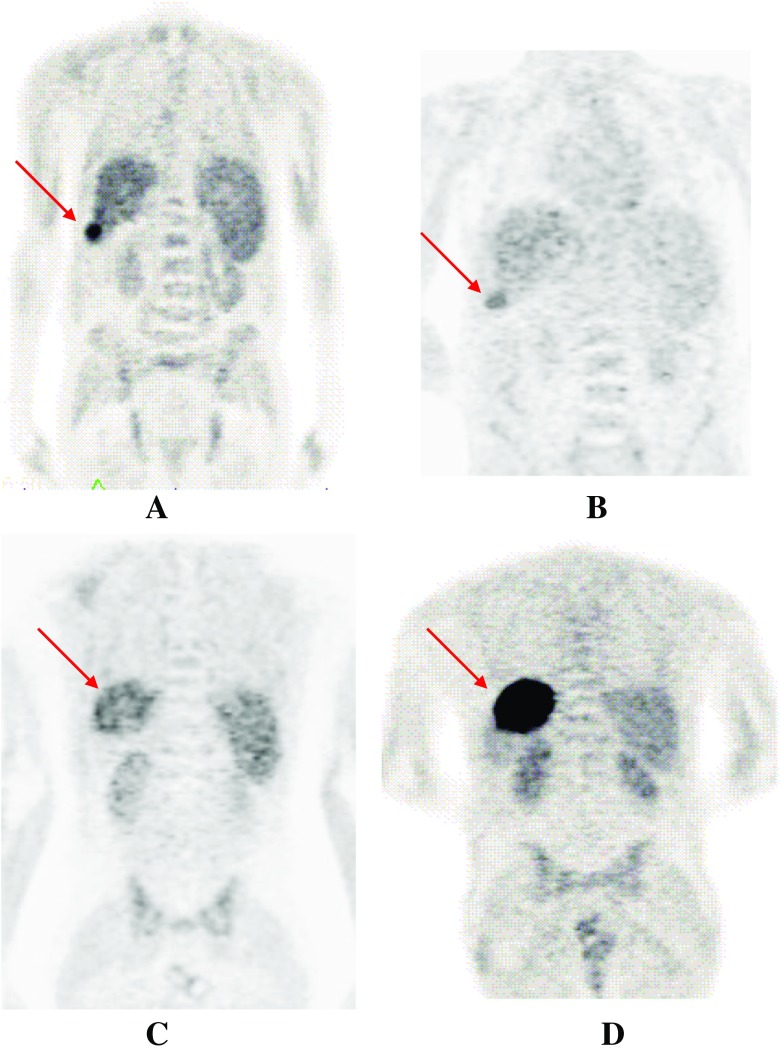
Images obtained from a patient with two HCC lesions having different tracer avidities. One lesion was strong positive for[^11^C]-acetate (A), but only weak positive for [^18^F]-FDG (B), whereas another lesion was strong positive for [^18^F]-FDG (D), but only weak positive for [^11^C]-acetate (C). The arrows indicate the two HCC lesions

Figure 2 showed in one patient with two HCC lesions having different tracer avidities. Before the treatment, one lesion in the segment VI of the liver was strong positive for [^11^C]-acetate (A), but only weak positive for [^18^F]-FDG (B), whereas another lesion in the segment VII was only weak positive for [^11^C]-acetate (C), but strong positive for [^18^F]-FDG (D). After the treatment with three cycles of TACE and six cycles of placebo, the two HCC lesions with heterogeneous tracer uptake demonstrating different response, the strong [^18^F]-FDG-avid lesion in the segment VII showing only partly response to the treatment, whereas the strong [^11^C]-acetate-avid lesion in the segment VI showing completely response to the treatment.

As shown in Table 2, there were significant differences in the tumor size changes after the treatment (*p* < 0.05). However, no significant differences were noted in the tumor size between patients treated with TACE + Bevacizumab and patients treated with TACE and placebo (*p* > 0.05).

**Fig. 3:**
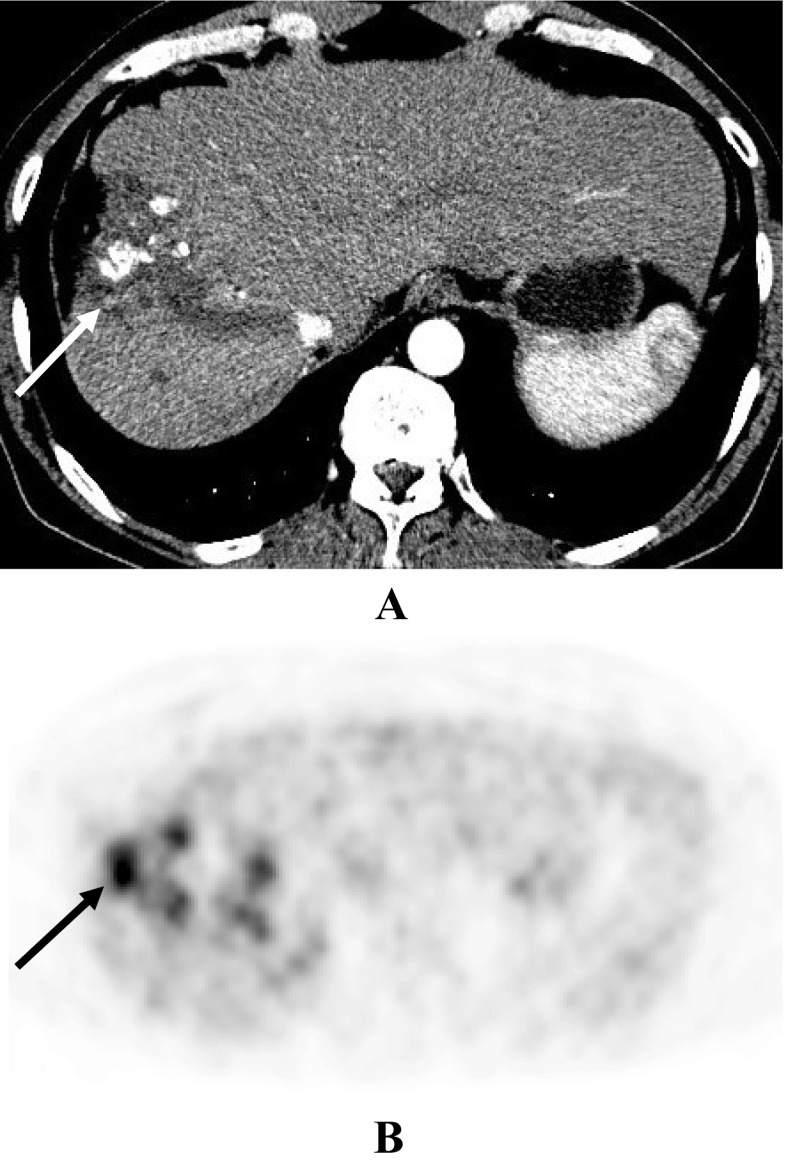
53-year-old man with partially necrotic HCC after TACE and placebo. (A), Transverse CT image (arterial phase) showing a subtotally necrotic tumor with low density masses and a focal hypervascularized tumor remnant (arrow) with patchy medium uptake; (B), Transverse [^18^F]-FDG PET showing high uptake of [^18^F]-FDG in the tumor remnant (arrow)

Figure 3 demonstrated a 53-year-old man with partially necrotic HCC after TACE and placebo. CT image revealed a partially necrotic tumor with low density masses and a focal hypervascularized tumor remnant (arrow) with patchy medium uptake (A); [^18^F]-FDG PET showed high uptake of [^18^F]-FDG in the tumor remnant (B).

### Patient follow-up

The mean duration of follow-up was 7.8 months. Twenty patients died of disease progression: eight within 6 months and 12 after 6 months. Two patients were still alive when follow-up was stopped for this study.

Comparison of the mean survival days between patients treated with TACE plus bevacizumab and patients with TACE plus placebo was shown in Table 3.

**Table 3:** Comparison of the mean survival days between patients treated with TACE plus bevacizumab and patients with TACE plus placebo

	TACE + Bevacizumab	TACE + Placebo	Means
	289 ± 190	479 ± 42	366 ± 341
patients with positive acetate PET	251 ± 231	408 ± 473	334 ± 376
patients with negative acetate PET	259 ± 118*	668 ± 217	434 ± 265
patients with positive FDG PET	309 ± 308	325 ± 294	318 ± 282
patients with negative FDG PET	322 ± 399	663 ± 515**	406 ± 391

*: *p* = 0.023 versus group TACE + Placebo; ^**^: *p* = 0.048 versus patients with positive FDG PET; Data are given as mean ± SD

In patients treated with TACE and bevacizumab, there were no significant differences (*p* > 0.136) in mean OS between the patients with positive acetate PET and the patients with negative acetate PET as well as between the patients with positive FDG PET and the patients with negative FDG PET.

In patients treated with TACE and placebo, there was a significant difference in mean OS in patients with positive FDG PET as compared with that in patients with negative FDG PET (*p* = 0.048). Although the OS days in patients with positive acetate PET were shorter as compared with those in patients with negative acetate PET, there was no statistically significant difference (*p* = 0.063).

Generally, the OS days in the TACE and bevacizumab group were shorter as compared with those in the TACE and placebo group. In the patients with negative acetate PET, the mean OS in TACE and bevacizumab group was significantly shorter as compared with that in TACE and placebo group (259 ± 118 days versus 668 + 217 days; *p* = 0.023).

## Discussion

Our present study explores the utility of combining [^18^F]-FDG and [^11^C]-acetate PET as a diagnostic or prognostic approach to HCC. The sensitivity of [^11^C]-acetate PET for the diagnosis of HCC in patients with HCC was higher than that of [^18^F]-FDG PET (68% versus 45%, respectively). The combination of the two tracers increased the sensitivity of PET to 73%, Therefore, the use of both [^18^F]-FDG and [^11^C]-acetate PET may be a valuable option to detect HCC. These findings are consistent with previous studies that radio-labeled acetate PET could be a valuable for detecting HCC and had greater diagnostic performance than [^18^F]-FDG PET [10]. The sensitivity of [^11^C]-acetate PET for the detection of HCC is in our study close to that reported by Park et al. [17], but is lower than that published by Hwang et al. [18]. They report a detection rate of 83% using [^11^C]-acetate and a sensitivity of 40% for [^18^F]-FDG PET, respectively. Other studies, such as that by Ho et al. report a sensitivity of 87% with [^11^C]-acetate and of 47% for [^18^F]-FDG [10], the combination of the two tracers increased the sensitivity of PET to 100% [10]. They conclude that performing PET with both radio-pharmaceuticals seemed to be the best diagnostic option.

In the present prospective study, we investigated the ability of [^18^F]-FDG and [^11^C]-acetate PET to assess metabolic response in HCC patients treated with TACE and bevacizumab compared with those in HCC patients treated with TACE and placebo. We found significantly higher acetate metabolic response rate in patients who were treated with TACE and bevacizumab than in patients treated with TACE and placebo. This indicates that bevacizumab may be more effective against HCC as compared with placebo. To the best of our knowledge, this is the first study regarding the assessment of the therapy response by using [^11^C]-acetate. Although the treatment with TACE and bevacizumab seems more effective than the therapy with TACE and placebo, the OS in patient group treated with TACE and bevacizumab is significantly shorter than that in the group treated with TACE and placebo [13]; this difference in survival seems to be mainly due to the significantly higher incidence of high-grade and even fatal vascular and septic side effects in patients treated with TACE and bevacizumab [13]. Bevacizumab has been known to cause several side effects including variceal bleeding, arterial hypertension and thrombosis [19, 20]. During this study, two fatal variceal bleeding events, several thromboembolic events, and one myocardial infarction, as well as abscess of the liver occurred only in the bevacizumab group (13). Our result found that the OS in the patient group treated with TACE and bevacizumab is significantly shorter than that in the group treated with TACE and placebo in all patients with negative acetate PET. This might have implication for clinical practice that patients with negative acetate PET should be more careful with the treatment with bevacizumab.

Several studies demonstrated that FDG-PET could better assess the tumor viability than CT after TACE [21] or radiofrequency ablation (RFA) [22] or transarterial selective internal radionuclide therapy (SIRT) with [^90^Y]-glass microsphere [23]. The results of our present study have also shown that the FDG metabolic response rate was also higher in the patients treated with TACE and bevacizumab than in patients treated with TACE and placebo. However, there was no statistically significant difference.

[^18^F]-FDG avidity appears to be more frequent in HCCs with a poor prognosis than in those with a good prognosis [24]. Several studies have shown that [^18^F]-FDG avidity is associated with an aggressive HCC phenotype, microvascular invasion [24], poor tumor differentiation [10, 25], and recurrence after surgical resection [26] or transplantation [27, 28]. Our study supports these previous findings. In the TACE and placebo group, patients with lesions showing [^18^F]-FDG uptake had significantly poorer prognosis than those with lesions that did not show [^18^F]-FDG uptake. Patients with positive [^11^C]-acetate lesions had also poor prognosis as compared with those with negative [^11^C]-acetate, which, however, did not reach the level of significance in our patient cohort. In patients treated with TACE and bevacizumab, there were no significant differences in the prognoses between the patients with positive acetate PET and the patients with negative acetate PET, as well as between the patients with positive FDG PET and the patients with negative FDG PET; this may due to the significantly higher incidence of side effects in this group of patients as compared with the TACE and placebo group. The present results demonstrated that uptake of [^18^F]-FDG or/and [^11^C]-acetate may be a marker of poor prognosis in patients. Recent study proposed [^11^C]-acetate having prognostic value for prostate cancer [29]. Our study has shown that in the TACE and placebo group, patients with the most unfavorable outcome during clinical follow-up were those with lesions showing [^18^F]-FDG or [^11^C]-acetate uptake. The results suggest that a negative PET may be associated with better overall survival and provide prognostic value beyond standard clinical information and CT-based anatomical staging.

Ho et al. [10] have reported that the well-differentiated HCC tumors are [^11^C]-acetate avidity and the poorly differentiated types are shown by [^18^F]-FDG. Interestingly, our study has shown that the poorly differentiated HCC are detected by both [^18^F]-FDG and [^11^C]-acetate and that some well differentiated HCCs are positive both in [^18^F]-FDG PET and [^11^C]-acetate PET. A few of tumors in patients with well differentiated HCC are detected by [^18^F]-FDG and many tumors in patients with well differentiated HCC were positive in [^11^C]-acetate PET. More interestingly, different tracer avidities were shown in one patient with two lesions in this study. These may be partly explained by the heterogeneity of HCC. We can assume that different grades of HCC lesions may exist in one patient. Hepatocellular carcinoma (HCC) is a highly heterogeneous from both clinical and molecular points of view [30]. However, there are very few reports on intra-tumor molecular heterogeneity in HCC in vivo. Our study showed the different tracer avidities in one patient with HCC lesions demonstrating the molecular heterogeneity in HCC. Aware of heterogeneity of HCC tumors is important since one of the key issues is to determine if this diversity significantly impacts predictions based on single biopsies, and ultimately, clinical decision making in the precision medicine. The potential clinical implications of this heterogeneity will also be pivotal to choose the effective molecular therapies and to understand resistance to molecular therapies.

This study has some limitations. One of the limitations is the size of the study population was modest, in which we had few poorly differentiated HCC lesions. Another limitation is that the choice of HCC treatment could also affect the clinical outcome. Indeed, as we reported earlier (13), patients who received TACE and bevacizumab had significantly higher incidence of high-grade and even fatal vascular and septic side effects as compared with the patients who were treated with TACE and placebo.

## Conclusion

Our study suggests that combining [^18^F]-FDG with [^11^C]-acetate PET could be useful for clinicians in the management of HCC patients. The use of dual-tracer PET might also provide relevant prognostic and molecular heterogeneity information. Thus, this approach might be valuable for the choose the effective molecular therapies and to identify patients who would most benefit from molecular therapies.
